# Synthesis and fluorosolvatochromism of 3-arylnaphtho[1,2-*b*]quinolizinium derivatives

**DOI:** 10.3762/bjoc.12.84

**Published:** 2016-05-02

**Authors:** Phil M Pithan, David Decker, Manlio Sutero Sardo, Giampietro Viola, Heiko Ihmels

**Affiliations:** 1 Department of Chemistry and Biology, University of Siegen and Center of Micro and Nanochemistry and Engineering, Adolf-Reichwein-Str. 2, 57068 Siegen, Germany; 2 University of Padova, Department of Pharmaceutical and Pharmacological Sciences, via Marzolo 5, 35131 Padova, Italy; 3 University of Padova, Department of Woman’s and Child’s health, 35128 Padova, Italy

**Keywords:** fluorescence, heterocycles, quinolizinium, solvatochromism

## Abstract

Cationic biaryl derivatives were synthesized by Suzuki–Miyaura coupling of 3-bromonaphtho[1,2-*b*]quinolizinium bromide with arylboronic acids. The resulting cationic biaryl derivatives exhibit pronounced fluorosolvatochromic properties. First photophysical studies in different solvents showed that the emission energy of the biaryl derivatives decreases with increasing solvent polarity. This red-shifted emission in polar solvents is explained by a charge shift (CS) in the excited state and subsequent solvent relaxation. Furthermore, the polarity of protic polar and aprotic polar solvents affects the emission energy to different extent, which indicates a major influence of hydrogen bonding on the stabilization of the ground and excited states.

## Introduction

Dyes that change their absorption and emission properties, especially their color, in different media are considered as helpful optical probes, because they may be applied for the characterization and identification of either bulk media or microscopic environments with relatively simple spectrometric analyses [1–3]. For example, distinct solvent properties, such as hydrogen-bonding ability, polarity or polarizability can be determined even quantitatively by means of solvatochromic optical probes [4–7]. In this context, fluorosolvatochromic probes appear to be especially attractive as indicators for the surrounding medium, because emission spectroscopy is a highly sensitive method that allows to determine three different physical quantities, namely emission quantum yield, emission energy and emission lifetime. Notably, a considerable number of solvatochromic molecules is based on charge-transfer (CT) processes in the excited state resulting from a pronounced donor–acceptor interplay within the fluorophore [8]. Thus, upon excitation of such compounds, a CT – or in charged species a charge shift (CS) – takes place that results in a significantly different electron distribution of the molecule in the excited state as compared to the ground state. As a consequence, the solvent molecules reorganize to re-establish an optimal stabilization of the molecules in the excited state, which is usually referred to as solvent relaxation. Thus, the different absorption and emission energies of solvatochromic compounds may result from different energies of the ground and excited state that are caused by the different stabilizing (or destabilizing) interactions between the solvent and the solutes [9–12].

**Figure 1:**
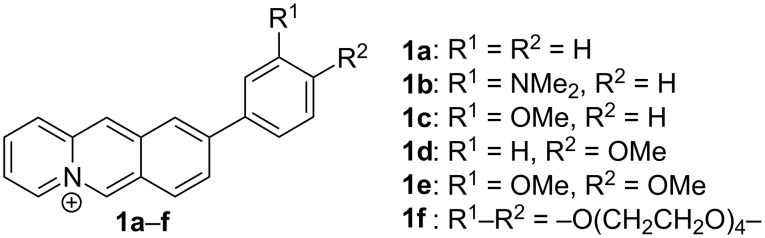
Structures of biaryl-type benzo[*b*]quinolizinium derivatives **1a**–**f**.

Along these lines, we established the annelated quinolizinium ion as a versatile platform for the investigation of cationic chemosensors, especially when the chromophore is incorporated within a donor–acceptor system [13]. In particular, we observed that biaryl-type quinolizinium derivatives such as **1a**–**f** (Figure 1) exhibit fluorosolvatochromic properties that are especially pronounced with donor-substituted aryl substituents [14–15].

In contrast, simple donor-substituted benzo[*b*]quinolizinium derivatives only show a relatively moderate solvatochromic behavior [16–17], which implies that the biaryl structure is an important feature that supports the solvatochromic behavior, most likely as it facilitates the CS in a twisted biaryl conformation [14–15]. To explore this property of annelated aryl-substituted quinolizinium derivatives further with a main focus on the fluorosolvatochromic probes, we developed novel 3-arylnaphtho[1,2-*b*]quinolizinium derivatives. Firstly, we chose the naphthoquinolizinium fluorophore because it should have essentially the same ability to act as an acceptor in the photoinduced CS process as the benzo[*b*]quinolizinium [18]. But due to its more extended π-system it may be better suited to delocalize the radical that is formed after the CS. The quinolizinium is further attached with unsubstituted aryl substituents, because it was shown that the latter may act as electron donating units in aryl-substituted acridinium and quinolinium derivatives [19–25]. We refrained from using additional donor functionalities such as the amino group, because we observed in a previous work that these substituents cause significant fluorescence quenching and lead to only weakly fluorescent derivatives [14]. Herein, we report the synthesis of novel 3-arylnaphtho[1,2-*b*]quinolizinium derivatives and demonstrate that some of these compounds have fluorosolvatochromic properties.

## Results

### Synthesis

**Scheme 1:**
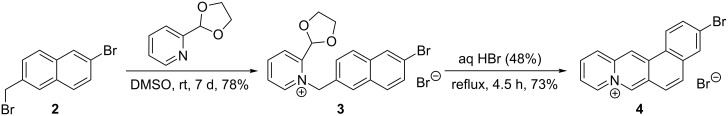
Synthesis of 3-bromonaphtho[1,2-*b*]quinolizinium bromide (**4**).

The 3-arylnaphtho[1,2-*b*]quinolizinium derivatives **6a**–**e** were prepared by Suzuki–Miyaura coupling reactions with the 3-bromonaphtho[1,2-*b*]quinolizinium bromide (**4**). The latter was synthesized from the known 2-bromo-6-(bromomethyl)naphthalene (**2**) which was prepared in two steps according to published procedures [26] from commercially available methyl 6-bromo-2-naphthoate. The reaction of the (bromomethyl)naphthalene **2** with (1,3-dioxolan-2-yl)pyridine yielded the *N*-benzylpyridinium derivative **3** and the subsequent cyclodehydration [27] in refluxing HBr (*w* = 48%) gave the bromonaphtho[1,2-*b*]quinolizinium **4** in 57% overall yield (Scheme 1).

**Scheme 2:**
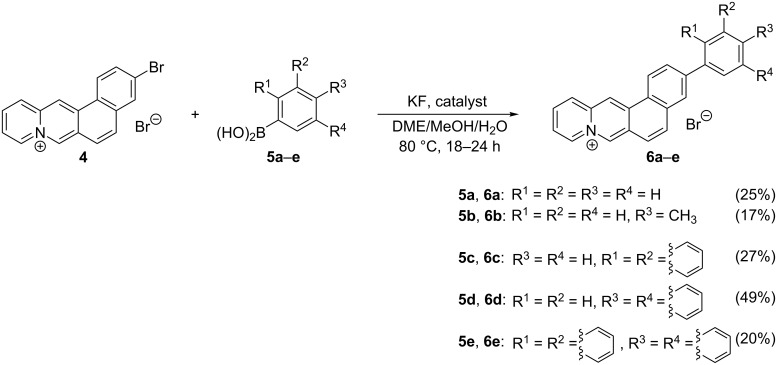
Synthesis of the 3-aryl-substituted naphtho[1,2-*b*]quinolizinium derivatives **6a**–**e**.

The Suzuki–Miyaura coupling reactions of 3-bromonaphthoquinolizinium derivative **4** with the arylboronic acids **5a**–**e** were performed under reaction conditions optimized for quinolizinium derivatives [28] with Pd(PPh_3_)_2_Cl_2_ or Pd(dppf)_2_Cl_2_·CH_2_Cl_2_ as catalyst and KF as mild base to give the respective aryl-substituted naphthoquinolizinium derivatives **6a**–**e** in 17–49% yield (Scheme 2). The structures of the new compounds **3**, **4** and **6a**–**e** were confirmed by NMR spectroscopic analysis (^1^H, ^13^C, COSY, HSQC, HMBC), mass-spectrometric data and elemental analysis.

### Absorption and emission properties

**Figure 2:**
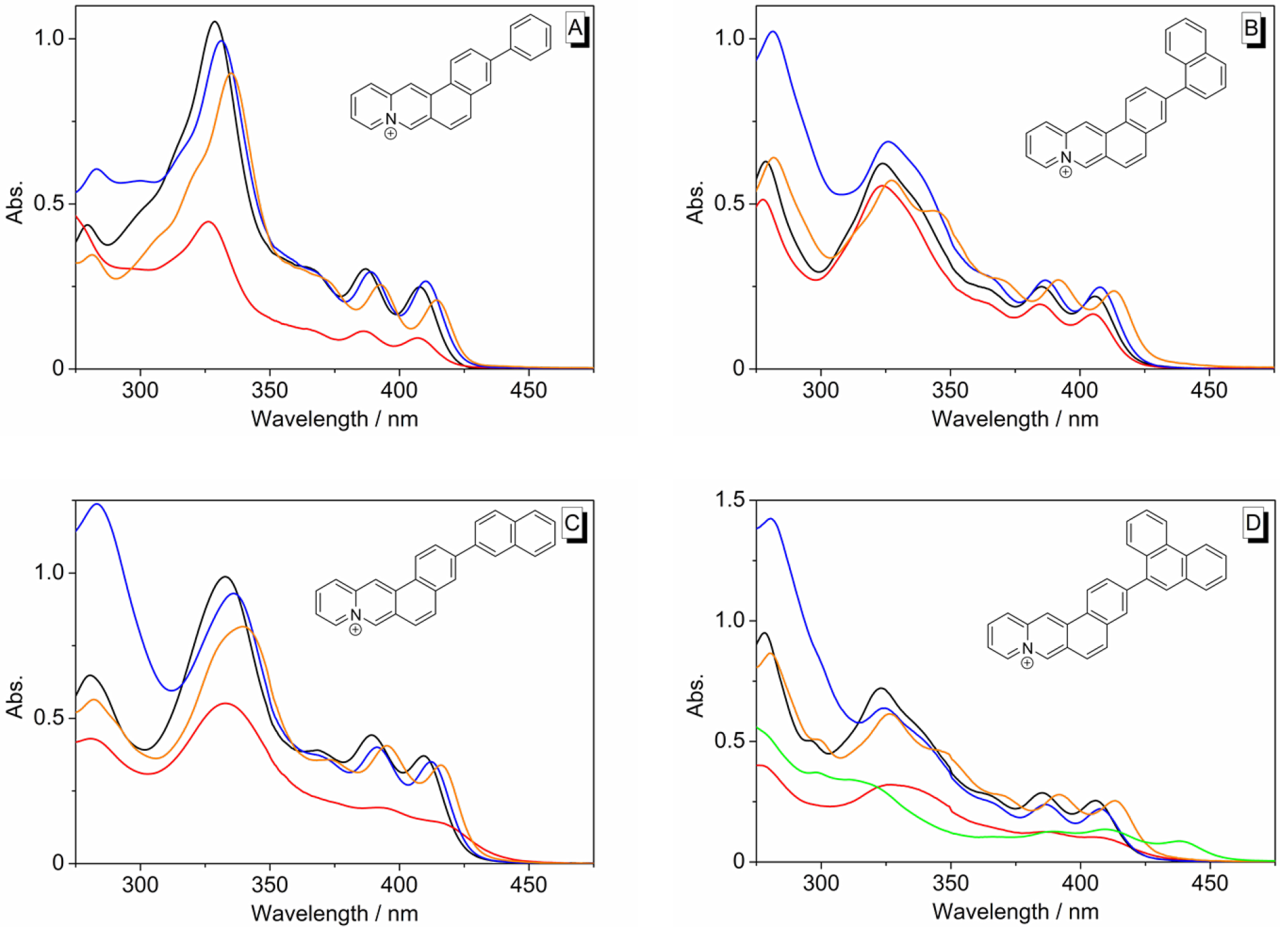
Absorption spectra of derivatives **6a** (A), **6c** (B), **6d** (C), and **6e** (D); *c* = 20 µM; solvents: H_2_O (red), MeOH (black), DMSO (blue), CHCl_3_ (orange), DME (green).

The 3-arylnaphthoquinolizinium derivatives **6a**–**e** are moderately soluble in protic polar and aprotic polar solvents and show the characteristic long-wavelength absorption band of the parent naphtho[1,2-*b*]quinolizinium [29] with two local maxima between 380 and 420 nm. The shifts of the absorption maxima of these compounds are almost independent from the solvent. For example, the long-wavelength absorption maximum of the phenyl-substituted derivative **6a** ranges from 407 nm in MeOH to 414 nm in CHCl_3_ (Table 1, Figure 2). As an exception, the absorption band of derivative **6e** in dimethoxyethane (DME) is significantly red-shifted (λ_abs_ = 438 nm). Notably, all compounds have particularly low extinction coefficients in H_2_O.

**Table 1:** Absorption and emission properties of quinolizinium derivatives **6a–e**.

	**6a**					**6b**			
			
Solvent^a^	λ_abs_^b^	lg ε^c^	λ_F_^d^	Φ_F_^e^ / 10^−2^		λ_abs_^b^	lg ε^c^	λ_F_^d^	Φ_F_^e^ / 10^−2^
		
H_2_O	408	3.67	421	34		407	3.91	471	53
MeOH	407	4.09	423	45		408	4.05	465	66
DMSO	410	4.13	425	3.4		411	4.16	456	6.0
CHCl_3_	414	4.02	428	1.4		415	4.09	455	3.1
									

	**6c**					**6d**			
			
Solvent^a^	λ_abs_^b^	lg ε^c^	λ_F_^d^	Φ_F_^f^ / 10^−2^		λ_abs_^b^	lg ε^c^	λ_F_^d^	Φ_F_^f^ / 10^−2^
		
H_2_O	405	3.92	546	1.9		416	3.84	524	19
MeOH	406	4.04	545	25		416	4.34	526	43
DMSO	408	4.09	559	17		412	4.24	538	18
CHCl_3_	413	4.07	473	4.2		416	4.23	470	3.4
									

	**6e**								
		
Solvent^a^	λ_abs_^b^	lg ε^c^	λ_F_^d^	Φ_F_^f^ / 10^−2^					
	
H_2_O	404	3.72	531	9.2					
MeOH	406	4.11	553	21					
EtOH	407	4.09	547	29					
AcOH	407	4.11	548	28					
BuOH	407	4.11	546	34					
2-PrOH	407	4.06	544	34					
MeCN	405	4.08	563	24					
DMSO	407	4.04	562	23					
Aceton	406	3.92	556	22					
CH_2_Cl_2_	411	4.04	538	17					
CHCl_3_	413	4.10	485	7.1					
DME	438	3.64	492	14					

^a^Solvents arranged in order of decreasing *E*_T_^30^ values. ^b^Long-wavelength absorption maximum in nm; *c* = 20 µM. ^c^Molar extinction coefficient in cm^–1^ M^–1^. ^d^Fluorescence emission maximum (Abs. = 0.10 at excitation wavelength); **6a**–**d**: λ_ex_ = 365 nm; **6e**: λ_ex_ = 385 nm. ^e^Fluorescence quantum yield relative to coumarin 1 [30–31]. ^f^Fluorescence quantum yield relative to coumarin 153 [30–31]; estimated error for fluorescence quantum yields: ±10%.

**Figure 3:**
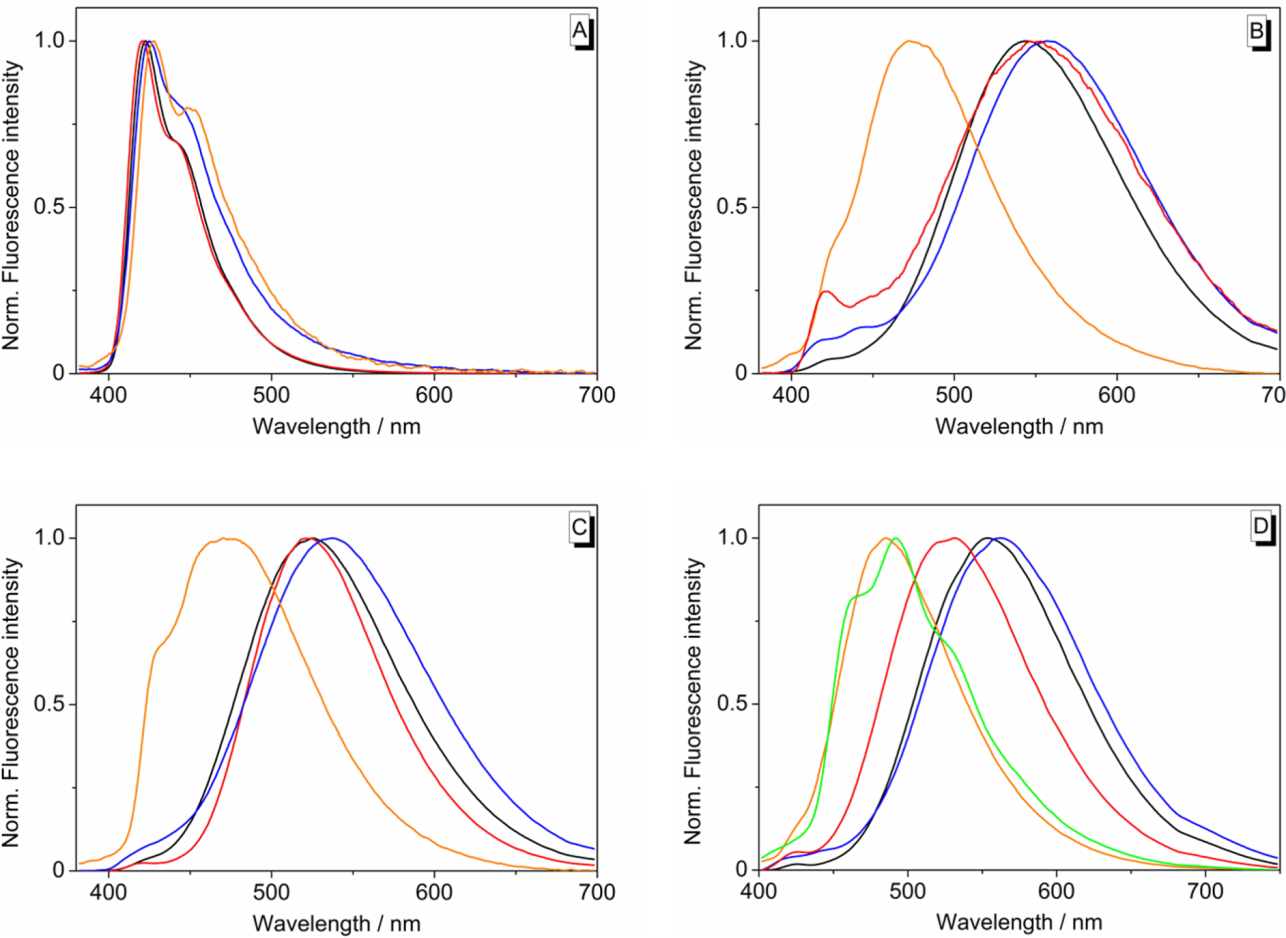
Normalized emission spectra of derivatives **6a** (A), **6c** (B), **6d** (C) and **6e** (D) (Abs. = 0.10 at excitation wavelength); **6a**, **6c**, **6d**: λ_ex_ = 365 nm; **6e**: λ_ex_ = 385 nm; solvents: H_2_O (red), MeOH (black), DMSO (blue), CHCl_3_ (orange), DME (green).

**Figure 4:**
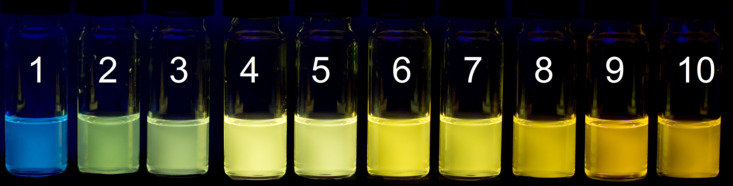
Fluorescence colors of derivative **6e** in various solvents; λ_ex_ = 366 nm. 1: CHCl_3_, 2: H_2_O, 3: CH_2_Cl_2_, 4: 1-BuOH, 5: 2-PrOH, 6: EtOH, 7: AcOH, 8: MeOH, 9: DMSO, 10: MeCN.

All derivatives **6a**–**e** are fluorescent. The emission maxima of the phenyl-substituted derivatives **6a** and **6b** deviate just slightly in different solvents (**6a**: 421–428 nm; **6b**: 455–471 nm). In contrast, the long-wavelength emission maxima of the naphthyl-substituted derivatives **6c** and **6d** vary, for instance, from 473 nm or 470 nm in CHCl_3_ to 559 nm or 538 in DMSO, respectively (Table 1, Figure 3A–D). Derivative **6e** is also fluorosolvatochromic, whereas the emission band is red-shifted by 78 nm from CHCl_3_ (λ_fl_ = 485 nm) to MeCN (λ_fl_ = 563 nm) (Figure 3D, Figure 4). Moreover, **6e** exhibits the highest Stokes shift in MeCN (Δλ = 63 000 cm^−1^). Unfortunately, the emission properties of **6a**–**d** in THF could not be determined due the very low solubility in this solvent. Derivate **6e** was moderately soluble in THF; however, it was not possible to record reproducible absorption and emission spectra due to slow partial decomposition of the compound in this solvent. For the derivatives **6a–d** the quantum yields were the highest in MeOH and the lowest in CHCl_3_. The phenanthrenyl-substituted derivative **6e** exhibits the highest fluorescence quantum yields in 2-PrOH and 1-BuOH (Φ_F_ = 0.34) and the lowest in CHCl_3_ (Φ_F_ = 0.071).

## Discussion

In most of the tested solvents the absorption maxima of the biaryl derivatives are only slightly red-shifted relative to the ones of the parent naphthoquinolizinium ion (e.g., λ_abs_ = 403 nm in MeOH) [32]. In the chloroalkane solvents CH_2_Cl_2_ and CHCl_3_ the red shift is, however, slightly more pronounced, which is commonly observed for cationic dyes and explained with the high polarizability of these solvents [33–34]. Furthermore, the derivative **6e** has a significantly red-shifted absorption in dimethoxyethane. Apart from these exceptional cases, however, the absorption properties depend only marginally on the solvent properties, such as the parent compound. This behavior indicates that the initial absorption does not contain a significant contribution of a charge-shift component and almost exclusively leads to the first local excited state (LE). The low extinction coefficients of all derivatives in water suggested aggregation in this solvent, as for example reported for 9-(4-dimethylaminophenyl)benzo[*b*]quinolizinium in aqueous solution [14]. Though, exemplary control experiments with derivative **6c** showed that the absorption of this compound depends linearly on the concentration (cf. Supporting Information File 1), which contradicts aggregation. Furthermore, the absorption band of **6c** is essentially maintained with increasing concentration. This behavior also indicates the absence of aggregates as the latter are characterized by the formation of red- or blue-shifted absorption bands or shoulders [35–36].

**Scheme 3:**
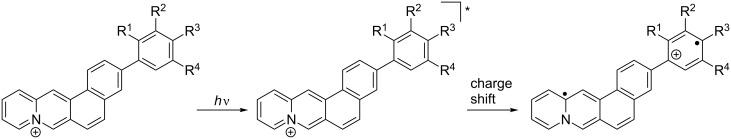
Intramolecular charge shift upon excitation in derivatives **6b**–**e** (see Scheme 2 for assignment of substituents).

The emission properties of the phenyl-substituted compound **6a** are also essentially independent of the solvent and resemble the fluorescence spectra of the parent naphthoquinolizinium (e.g., λ_F_ = 420 nm in MeOH) [34], which indicates emission from the LE state without specific stabilization or destabilization of the excited molecule after solvent relaxation. In contrast, the biaryl derivatives **6b**–**e** have significantly red-shifted emission bands, especially in more polar solvents. Notably, this effect is more pronounced with increasing ring size and for that matter with the electron-donating ability of the substituent (tolyl < naphthyl ≈ phenanthryl). Such a red shift of the emission bands was already observed for acridinium- and benzo[*b*]quinolizinium-containing biaryl derivatives and shown to result from a photoinduced electron transfer from the electron-donating aryl unit to the excited cationic hetarene, thus leading to a charge shift (CS) in the excited state [19–25]. Thus, the results of the absorption and steady-state emission experiments of compounds **6b**–**e**, along with literature precedence, point to an initial excitation of the ground state molecule to the LE state, followed by a charge shift (CS) to generate an intermediate excited species, that is a combination of charge neutral radical, i.e., a quinolizinyl radical, and the radical cation of the aromatic substituent (Scheme 3). The proposed photoinduced charge shift in derivatives **6b**–**e** is supported by observations that even the *inter*molecular electron transfer reactions between electron rich aromatic compounds, such as naphthalene and phenanthrene, and the excited benzo[*b*]quinolizinium fluorophore were shown to be efficient processes [37].

**Figure 5:**
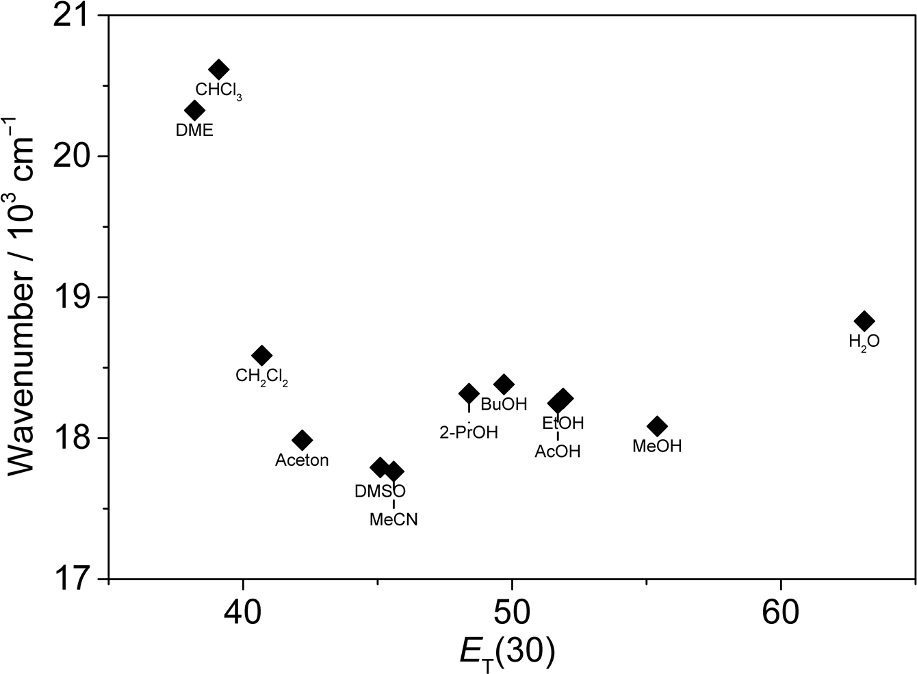
Plot of the emission energy of **6e** versus the solvent polarity parameter *E*_T_(30).

The biaryl derivatives **6b**–**e** exhibit fluorosolvatochromism that is characteristic of donor–acceptor dyes, namely a cumulative red shift of the emission maximum with increasing solvent polarity, along with a broad, unstructured band structure [1–2]. To assess the effect of the solvent on the emission of compound **6e** several different solvents were employed. Unfortunately, many solvents – mainly less polar or non-polar – could not be used because of the limited solubility of the analyte. Remarkably, neither the emission maxima, nor the Stokes shifts correlate well with common solvent parameters, such as the hydrogen bond (HB) donating properties, α, HB accepting properties, β, the Taft parameter π*, the dipole moment, μ, the acidity, SA, the basicity, SB, the dipolarity and polarizability S_PP_, the polarizability, SP, or the dipolarity SDP (cf. Supporting Information File 1). In all cases, the plots of the solvent parameters versus the emission energy do not disclose an obvious relationship. This behavior is in agreement with that of other donor–acceptor biaryl-type dyes, such as for example *para*-hetaryl-substituted benzophenone derivatives, whose emission properties were demonstrated to depend on a complex interplay of different solvent parameters [38]. Unfortunately, the limited solubility of the derivatives **6b**–**e** did not permit to employ a large series of solvents that is required for such a multiparameter analysis of these compounds. Nevertheless, the separation of the solvents into protic and aprotic solvents reveals at least a general trend of the solvent effect on the emission properties of **6e**. Namely, the emission maxima shift to lower energy with increasing polarity of the aprotic solvents (Figure 5). This qualitative relationship denotes a larger dipole moment of the molecule in the excited state than in the ground state, as usually observed in donor–acceptor dyes [3]. However, the linear regression analysis of a plot of the emission energy versus solvent polarity, as quantified by the *E*_T_(30) value, did not produce a good correlation, which indicates that solvent properties other than the polarity also contribute to some extent to the stabilization or destabilization of the ground and/or excited state of **6e**.

**Scheme 4:**
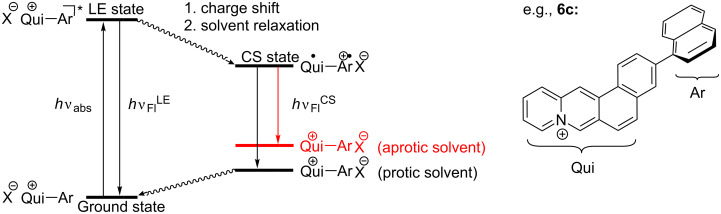
State diagram of the photoexcitation and deactivation pathways of 3-aryl-naphthoquinolizinium derivatives.

Of special note are the different emission properties of **6e** in protic and aprotic polar solvents (Figure 5). In particular, these two classes of solvents differ in their hydrogen bond (H-bond) donating properties. The latter usually stabilize negative polarized or charged species, which is most likely the bromide counter ion in the case of the salt **6e**. In fact, the fate of the counter ion during the photoinduced CS in cationic biaryl derivatives was already discussed [24,39]. Specifically, it was proposed that after the CS, and likely during solvent relaxation, the counter anion migrates to the radical cation unit at the aryl substituent. At the same time, this mechanism implies that directly after the "back CS" the bromide anion is still located in the vicinity of the aryl substituent and therefore no longer compensated by a nearby cationic charge before it moves back to the cationic hetarene in a subsequent relaxation process (Scheme 4). In protic solvents, the bromide ion is still stabilized by H-bonding. In contrast, in aprotic polar solvents the anion cannot be stabilized sufficiently which leads to an increased energy of this state. As a result the emission from the CS state in aprotic solvents has a lower energy and exhibits an emission maximum that is red-shifted relative to the emission in protic solvents.

**Figure 6:**
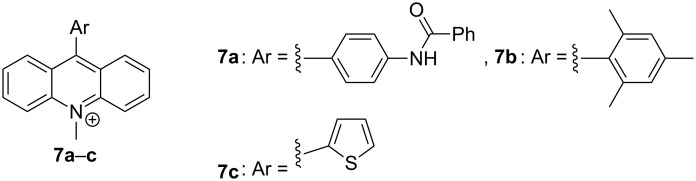
Structures of biaryl derivatives **7a**–**c**.

Unfortunately, these results did not enable a quantitative assessment of the solvent parameters that govern the steady-state emission energy of biaryl-type quinolizinium derivatives **6a**–**e**; but the most relevant solvent properties were identified that cause the fluorosolvatochromism of these derivatives. Interestingly, attempts to compare these results with the ones of related aryl-substituted cationic hetarenes and to draw a consistent picture of the solvatochromism of this class of compounds turned out to be rather difficult. Specifically, different cationic biaryl derivatives (Figure 6) show significantly varying trends regarding the influence of the solvent on the steady-state emission. For example, it was reported that the emission maximum of the CS emission of (benzoylamino)phenyl-10-methylacridinium (**7a**) is shifted to lower energy in solvents with decreasing polarity of the solvent, presumably as in this case the ground state dipole is larger than the one in the excited state [40]. In contrast, however, it was stated that there is no significant difference between the ground and excited-state dipole of the 9-mesityl-10-methylacridinium (**7b**), because the emission energies do not correlate with the Lippert–Mataga solvent parameter [41]. Furthermore, the 9-thienyl-10-methylacridinium (**7c**) is not solvatochromic at all, which was explained by a similar delocalization of the charge in the ground and excited state [21]. Overall, these results, along with ones observed in this work, clearly show that the steady-state fluorosolvatochromism of biaryl-type cationic hetarenes depends on a very fine balance of the donor–acceptor interplay in the ground and excited state, specifically the resulting delocalization of the charge. Unfortunately, detailed investigations of the steady-state solvatochromism of this class of compounds are rather scarce, so far, as most photophysical studies of 9-aryl-9-methylacridinium or 4-aryl-*N*-methylquinolinium have a strong emphasis on the dynamics and the charge separation in the excited state. Therefore, the work presented here may initiate investigations along these lines to further understand and fully explore the useful solvatochromic properties of this class of compounds.

## Conclusion

In summary, we present a new class of solvatochromic cationic biaryl derivatives whose emission properties depend strongly on the solvent properties. The synthesis of these compounds is straightforward, and variations of substrate structures should allow the synthesis of further derivatives. First photophysical studies showed that the emission energy of the biaryl derivatives **6b**–**e** is progressively red shifted with increasing polarity of the solvent, which is explained by a charge shift in the excited state and subsequent solvent relaxation. Notably, the polarity of protic polar and aprotic polar solvents affects the emission energy to a different degree, thus denoting a major influence of hydrogen bonding on the stabilization of the ground and excited states. Based on these results it is concluded that the solvent-sensitive emission properties of biaryl-type quinolizinium fluorophores are a promising structural motif for the development of novel solvatochromic probes.

## Supporting Information

The Supporting Information contains the experimental section (synthesis, determination of fluorescence quantum yields); absorption and emission spectra of **6b**; ^1^H NMR spectra of compounds **6a**–**e**; plots of emission energies of **6e** versus selected solvent parameters; plot of the absorbance of **6c** in H_2_O at λ_abs_ = 405 nm versus concentration.

File 1 Experimental and analytical data.
