# Supplementary material for: Synthesis and fluorosolvatochromism of 3-arylnaphtho[1,2-b]quinolizinium derivatives
Source: Beilstein J Org Chem. 2016 May 2;12:854–62. doi: 10.3762/bjoc.12.84 (PMC4901894; doi:10.3762/bjoc.12.84)
Supplement: File 1 — Experimental and analytical data. [file Beilstein_J_Org_Chem-12-854-s001.pdf]

Supporting Information

for

Synthesis and fluorosolvatochromism of 3-arylnaphtho[1,2-*b*]quinolizinium derivatives

Phil M. Pithan,[1] David Decker,[1] Manlio Sutero Sardo,[2] Giampietro Viola[3] and Heiko Ihmels[1]*

Address: [1]Department of Chemistry and Biology, University of Siegen and Center of Micro and Nanochemistry and Engineering, Adolf-Reichwein-Str. 2, 57068 Siegen (Germany), [2]University of Padova, Department of Pharmaceutical and Pharmacological Sciences, via Marzolo 5, 35131 Padova (Italy) and [3] University of Padova, Department of Woman's and Child's health, 35128 Padova (Italy)

Email: Heiko Ihmels - ihmels@chemie.uni-siegen.de

*Corresponding author

Experimental and analytical data

Table of contents

1 Experimental Section s2

1.1 General s2

1.2 Synthesis s2

1.3 Determination of fluorescence quantum yields s6

2 Additional spectroscopic data s8

3 ^{1}H NMR spectra of derivatives 3, 4 and 6a–e s10

4 References s14

1 Experimental Section

1.1 General

NMR spectra were recorded with a Bruker Avance 400 (^{1}H: 400 MHz, ^{13}C: 100 MHz) at room temperature (approximately 22 °C) or with a Varian VNMR-S 600 (^{1}H: 600 MHz, ^{13}C: 150 MHz) at 25 °C or 35 °C (DMSO-d_6). Spectra were analysed with the software ACD/NMR Processor Academic Edition 12.02 and referenced to the solvent DMSO-d_6 (^{1}H = 2.50, ^{13}C = 39.5). The chemical shifts are given in ppm. Absorption spectra were recorded with a Cary 100 bio spectrophotometer in quartz cells (10 mm x 10 mm) with baseline correction. Emission spectra were collected in quartz cells (10 mm x 10 mm) with a Cary Eclipse spectrophotometer at 20 °C. Elemental analyses data were determined with a HEKAtech EUROEA combustion analyzer by Mr. Rochus Breuer (Universität Siegen, Organische Chemie I). Mass spectra (ESI) were recorded on a Finnigan LCQ Deca (U = 6 kV; working gas: argon; auxiliary gas: nitrogen; temperature of the capillary: 200 °C). The melting points were measured with a Büchi 545 (Büchi, Flawil, CH) and they are uncorrected.

1.2 Synthesis

*1-[(6-Bromonaphth-2-yl)methyl]-2-(1,3-dioxolan-2-yl)pyridinium bromide (**3**)*

A solution of 2-bromo-6-(bromomethyl)naphthalene (**2**) (2.38 g, 7.99 mmol) and 2-(1,3-dioxolane-2-yl)pyridine (1.33 g, 8.80 mmol) in DMSO (60 mL) was stirred under an inert gas atmosphere for 7 d at room temperature. The reaction mixture was added dropwise under vigorous stirring to EtOAc (1 L). The white precipitate was filtered, washed with EtOAc (50 mL) and Et_2O (50 mL) and dried in vacuo to give the pure product as white amorphous solid which was used without further purification (2.81 g, 6.23 mmol, 78%). For analytical purposes a sample was recrystallized from MeOH/EtOAc to give the product **3** as bright beige-colored microcrystalline solid; mp = 193–195 °C (dec.). – ^{1}H-NMR (400 MHz, DMSO-d_6): δ = 4.11–4.13 (m, 4 H, 2 x CH_2), 6.16 (s, 2 H, CH_2), 6.56 (s, 1 H, CH), 7.55 (dd, ^{3}J = 8.8 Hz, ^{4}J = 1.8 Hz, 1 H, 3-H), 7.69 (dd, ^{3}J = 8.8 Hz, ^{4}J = 2.0 Hz, 1 H, 7-H), 7.87 (s, 1 H, 1-H), 7.90 (d, ^{3}J = 8.8 Hz, 1 H, 8-H), 7.99 (d, ^{3}J = 8.8 Hz, 1 H, 4-H), 8.20–8.23 (m, 1 H, 5'-H), 8.27 (d, ^{4}J = 1.8 Hz, 1 H, 5-H), 8.36 (dd, ^{3}J = 7.9 Hz, ^{4}J = 1.2 Hz, 1 H, 3'-H), 8.73–8.77 (m, 1 H, 4'-H), 9.11 (dd, ^{3}J = 6.1 Hz, ^{4}J = 0.9 Hz, 1 H, 6'-H). – ^{13}C-NMR (100 MHz, DMSO-d_6): δ = 59.9 (CH_2), 65.7 (2 x CH_2), 97.2 (CH), 120.2 (6-C), 126.1 (3'-C), 126.6 (3-C), 127.5 (1-C), 128.1 (4-C), 128.7 (5'-C), 129.6 (5-C), 129.8 (7-C), 130.3 (8-C), 131.2 (8a-C), 132.1 (2-C), 133.9 (4a-C), 147.3 (4'-C), 147.4 (6'-C), 152.2 (2'-C). – MS (ESI^{+}): m/z = 370 (100) $[M-Br]^{+}$ – El. Anal. for $C_{19}H_{17}Br_2NO_2$ (451.16), calcd (%): C 50.58, H 3.80, N 3.10, found (%): C 50.54, H 3.75, N 3.04.

*3-Bromonaphtho[1,2-b]quinolizinium bromide (**4**)*

A solution of pyridinium derivative **3** (2.80 g, 6.21 mmol) in aq. HBr (48%, 28 mL) was stirred under reflux for 4.5 h. After cooling to room temperature, THF (50 mL) was added to the reaction mixture and the orange-colored precipitate was collected, washed with THF (25 mL) and Et_2O (25 mL). The analytically pure product **4** was obtained by two-fold recrystallization from MeOH as fine shiny yellow needles (1.75 g, 4.49 mmol, 73%); mp > 300 °C (dec.). – ^{1}H-NMR (600 MHz, DMSO-d_6): δ = 8.08–8.10 (m, 1 H, 10-H), 8.10 (dd, ^3J = 8.8 Hz, ^4J = 2.4 Hz, 1 H, 2-H), 8.13 (br. s, 2 H, 5-H, 6-H), 8.27 (ddd, ^3J = 7.6 Hz, ^3J = 6.8 Hz, ^4J = 0.8 Hz, 1 H, 11-H), 8.43 (d, ^4J = 2.4 Hz, 1 H, 4-H), 8.64 (d, ^3J = 8.8 Hz, 1 H, 12-H), 8.99 (d, ^3J = 8.8 Hz, 1 H, 1-H), 9.40 (d, ^3J = 6.8 Hz, 1 H, 9-H), 10.05 (s, 1 H, 13-H), 10.29 (s, 1 H, 7-H). – ^{13}C-NMR (150 MHz, DMSO-d_6): δ = 120.7 (13-C), 122.9 (10-C), 124.7 (6-C), 125.4 (3-C, 6a-C), 125.6 (13b-C), 126.9 (12-C), 127.1 (1-C), 131.3 (5-C), 131.5 (4-C), 131.7 (2-C), 133.3 (11-C), 134.7 (9-C), 134.7 (13a-C), 134.9 (4a-C), 137.5 (7-C), 138.9 (12a-C). – MS (ESI$^+$): *m*/*z* = 308 (100) $[M-Br]^+$. – El. Anal. for $C_{17}H_{11}Br_2N$ x H_2O (407.11), calcd (%): C 50.16, H 3.22, N 3.44, found (%): C 50.10, H 3.23, N 3.48.

General procedure for the Suzuki–Miyaura coupling of 3-bromonaphtho[1,2-*b*]quinolizinium bromide with arylboronic acids (GP 1)[1]

Under an inert gas atmosphere, a suspension of 3-bromonaphtho[1,2-*b*]quinolizinium bromide (**4**) (1.00 mmol), the corresponding arylboronic acid (1.10 mmol), $Pd(PPh_3)_2Cl_2$ (30.0 µmol) or $Pd(dppf)_2Cl_2 \cdot CH_2Cl_2$ (30.0 µmol) and KF (4.00 mmol) in DME/MeOH/H_2O (2/1/1; 12 mL) was stirred at 80 °C for 18–24 h (reaction monitored by TLC). MeOH (20 mL) was added to the reaction mixture and the precipitated palladium-black was removed by hot filtration. After cooling the filtrate to room temperature, the yellow precipitate was collected, washed with EtOAc and Et_2O (5 mL). The analytically pure product was isolated by recrystallization of the precipitate from MeOH.

*3-Phenylnaphtho[1,2-b]quinolizinium bromide (**6a**)*

According to GP 1, the naphthoquinolizinium **4** (389 mg, 1.00 mmol) was made to react with phenyl boronic acid (**5a**) (134 mg, 1.10 mmol) for 20 h using $Pd(PPh_3)_2Cl_2$ (21.1 mg, 30.0 µmol) as catalyst. The product **6a** was obtained as dark orange colored, microcrystalline solid (97.3 mg, 252 µmol, 25%); mp > 300 °C. – ^{1}H-NMR (600 MHz, DMSO-d_6): δ = 7.49–7.52 (m, 1 H, 4'-H), 7.58–7.61 (m, 2 H, 3'-H, 5'-H), 7.96–7.98 (m, 2 H, 2'-H, 6'-H), 8.04–8.07 (m, 1 H, 10-H), 8.15 (d, ^3J = 8.8 Hz, 1 H, 6-H), 8.24–8.29 (m, 2 H, 5-H, 11-H), 8.33 (dd, ^3J =

8.4 Hz, ^4J = 2.0 Hz, 1 H, 2-H), 8.54 (d, ^4J = 2.0 Hz, 1 H, 4-H), 8.65 (d, ^3J = 8.8 Hz, 1 H, 12-H), 9.18 (d, ^3J = 8.8 Hz, 1 H, 1-H), 9.37 (d, ^3J = 6.8 Hz, 1 H, 9-H), 10.08 (s, 1 H, 13-H), 10.26 (s, 1 H, 7-H). – ^{13}C-NMR (150 MHz, DMSO-d_6): δ = 120.4 (13-C), 122.6 (10-C), 123.6 (6-C), 125.7 (6a-C),125.8 (1-C), 125.8 (13b-C), 126.8 (12-C), 126.9 (4-C), 127.2 (2'-C, 6'-C), 127.4 (2-C), 128.6 (4'-C), 129.1 (3'-C, 5'-C), 132.8 (5-C), 132.9 (11-C), 134.0 (4a-C), 134.6 (9-C), 135.1 (13a-C), 137.3 (7-C), 138.4 (1'-C), 138.8 (12a-C), 143.0 (3-C). – MS (ESI$^+$): *m/z* = 306 (100) [M–Br]$^+$. – El. Anal. for $C_{23}H_{16}BrN$ (385.9), calcd (%): C 71.51, H 4.18, N 3.63, found (%): C 71.68, H 4.10, N 3.60.

*3-(4-Methylphenyl)naphtho[1,2-b]quinolizinium bromide (**6b**)*

According to GP 1, the naphthoquinolizinium **4** (389 mg, 1.00 mmol) was made to react with 4-methylphenyl boronic acid (**5b**) (150 mg, 1.10 mmol) for 20 h using $Pd(PPh_3)_2Cl_2$ (21.1 mg, 30.0 µmol) as catalyst. The product **6b** was obtained as dark yellow fine needles (67.4 mg, 168 µmol, 17%); mp > 300 °C. – ^{1}H-NMR (600 MHz, DMSO-d_6): δ = 2.41 (s, 3 H, CH_3), 7.38–7.40 (m, 2 H, 3'-H, 5'-H), 7.85–7.87 (m, 2 H, 2'-H, 6'-H), 8.03–8.05 (m, 1 H, 10-H), 8.12 (d, ^3J = 8.8 Hz, 1 H, 6-H), 8.23–8.26 (m, 2 H, 5-H, 11-H), 8.28 (dd, ^3J = 8.4 Hz, ^4J = 2.0 Hz, 1 H, 2-H), 8.49 (d, ^4J = 2.0 Hz, 1 H, 4-H), 8.63 (d, ^3J = 8.8 Hz, 1 H, 12-H), 9.12 (d, ^3J = 8.8 Hz, 1 H, 1-H), 9.35 (d, ^3J = 6.8 Hz, 1 H, 9-H), 10.05 (s, 1 H, 13-H), 10.25 (s, 1 H, 7-H). – ^{13}C-NMR (150 MHz, DMSO-d_6): δ = 20.8 (4'-CH_3), 120.3 (13-C), 122.6 (10-C), 123.7 (6-C), 125.6 (13b-C), 125.7 (6a-C), 125.8 (1-C), 126.5 (4-C), 126.9 (12-C), 127.1 (2'-C, 6'-C), 127.2 (2-C), 129.8 (3'-C, 5'-C), 132.8 (5-C), 133.0 (11-C), 134.1 (4a-C), 134.7 (9-C), 135.2 (13a-C), 135.5 (1'-C), 137.4 (7-C), 138.3 (4'-C), 138.9 (12a-C), 142.9 (3-C). – MS (ESI$^+$): *m/z* = 320 (100) [M–Br]$^+$. – El. Anal. for $C_{24}H_{18}BrN$ (400.3), calcd (%): C 72.01, H 4.53, N 3.50, found (%): C 71.84, H 4.48, N 3.52.

*3-(Naphth-1-yl)naphtho[1,2-b]quinolizinium bromide (**6c**)*

According to GP 1, the naphthoquinolizinium **4** (389 mg, 1.00 mmol) was made to react with naphthalene-1-boronic acid (**5c**) (189 mg, 1.10 mmol) for 20 h using $Pd(PPh_3)_2Cl_2$ (21.1 mg, 30.0 µmol) as catalyst. In deviation from GP 1, the solvent of the filtrate was removed *in vacuo*, the residue was redissolved in DMSO (2 mL) and added dropwise to EtOAc (150 mL). The yellow precipitate was collected, washed with H_2O (5 mL), EtOAc (2 x 20 mL) and Et_2O (2 x 20 mL). The analytically pure product **6c** was separated by two-fold recrystallization of the precipitate from MeOH and obtained as ocher-colored, amorphous solid (117 mg, 268 µmol, 27%); mp > 300 °C. – ^{1}H-NMR (600 MHz, DMSO-d_6): δ = 7.55–7.57 (m, 1 H, 7'-H), 7.60–7.63 (m, 1 H, 6'-H), 7.65 (dd, ^3J = 6.8 Hz, ^4J = 1.2 Hz, 1 H, 2'-H), 7.68–7.70 (m, 1 H, 3'-H), 7.89 (d, ^3J = 8.4 Hz, 1 H, 8'-H), 8.08–8.11 (m, 3 H, 10-H, 4'-H, 5'-H), 8.13 (dd, ^3J = 8.0 Hz, ^4J = 1.6 Hz, 1 H, 2-H), 8.19 (d, ^3J = 8.8 Hz, 1 H, 6-H), 8.27–8.29 (m, 1 H, 11-H), 8.31

(d, ^3J = 9.2 Hz, 1 H, 5-H), 8.36 (d, ^4J = 2.0 Hz, 1 H, 4-H), 8.69 (d, ^3J = 8.8 Hz, 1 H, 12-H), 9.27 (d, ^3J = 8.8 Hz, 1 H, 1-H), 9.42 (d, ^3J = 6.8 Hz, 1 H, 9-H), 10.17 (s, 1 H, 13-H), 10.33 (s, 1 H, 7-H). – ^{13}C-NMR (150 MHz, DMSO-d_6): δ = 120.6 (13-C), 122.8 (10-C), 123.8 (6-C), 125.0 (8'-C), 125.4 (1-C), 125.6 (3'-C), 125.8 (6a-C), 125.9 (13b-C), 126.2 (6'-C), 126.9 (7'-C), 127.0 (12-C), 127.6 (2'-C), 128.6 (4'-C, 5'-C), 130.4 (4-C), 130.6 (8a'-C), 130.8 (2-C), 132.7 (5-C), 133.1 (11-C), 133.5 (4a'-C), 133.7 (4a-C), 134.8 (9-C), 135.3 (13a-C), 137.5 (7-C), 138.1 (1'-C), 139.0 (12a-C), 143.4 (3-C). – MS (ESI$^+$): *m*/*z* = 356 (100) [M–Br]$^+$. – El. Anal for $C_{27}H_{18}BrN$ x H_2O (454.4), calcd (%): C 71.37, H 4.44, N 3.08, found (%): C 71.38, H 4.23, N 3.07.

*3-(Naphth-2-yl)naphtho[1,2-b]quinolizinium bromide (**6d**)*

According to GP 1, the naphthoquinolizinium **4** (389 mg, 1.00 mmol) was made to react with naphthalene-2-boronic acid (**5d**) (189 mg, 1.10 mmol) for 18 h using $Pd(dppf)_2Cl_2 \cdot CH_2Cl_2$ (24.5 mg, 30.0 µmol) as catalyst. The product **6d** was obtained as yellow, amorphous solid (214 mg, 490 µmol, 49%); mp > 300 °C. – ^{1}H-NMR (600 MHz, DMSO-d_6): δ = 7.58–7.63 (m, 2 H, 6'-H, 7'-H), 8.00–8.01 (m, 1 H, 5'-H), 8.04–8.06 (m, 1 H, 10-H), 8.08–8.09 (m, 1 H, 8'-H), 8.12 (br. s, 2 H, 3'-H, 4'-H), 8.16 (d, ^3J = 8.8 Hz, 1 H, 6-H), 8.24–8.27 (m, 1 H, 11-H), 8.32 (d, ^3J = 9.2 Hz, 1 H, 5-H), 8.47 (dd, ^3J = 8.4 Hz, ^4J = 2.0 Hz, 1 H, 2-H), 8.54 (br. s, 1 H, 1'-H), 8.65 (d, ^3J = 8.4 Hz, 1 H, 12-H), 8.68 (d, ^4J = 2.0 Hz, 1 H, 4-H), 9.21 (d, ^3J = 8.4 Hz, 1 H, 1-H), 9.36 (d, ^3J = 6.8 Hz, 1 H, 9-H), 10.08 (s, 1 H, 13-H), 10.26 (s, 1 H, 7-H). – ^{13}C-NMR (150 MHz, DMSO-d_6): δ = 120.4 (13-C), 122.6 (10-C), 123.7 (6-C), 124.9 (3'-C), 125.7 (6a-C), 125.8 (13b-C), 125.9 (1-C), 126.3 (1'-C), 126.6 (7'-C), 126.7 (6'-C), 126.9 (12-C), 127.2 (4-C), 127.5 (5'-C), 127.6 (2-C), 128.3 (8'-C), 128.7 (4'-C), 132.7 (4a'-C), 132.8 (5-C), 132.9 (11-C), 133.2 (8a'-C), 134.1 (4a-C), 134.7 (9-C), 135.2 (13a-C), 135.7 (2'-C), 137.4 (7-C), 138.9 (12a-C), 142.8 (3-C). – MS (ESI$^+$): *m*/*z* = 356 (100) [M–Br]$^+$. – El. Anal. for $C_{27}H_{18}BrN$ x H_2O (454.4), calcd (%): C 71.37, H 4.44, N 3.08, found (%): C 71.26, H 4.01, N 3.08.

*3-(Phenanthrene-9-yl)naphtho[1,2-b]quinolizinium bromide (**6e**)*

According to GP 1, the naphthoquinolizininum **4** (195 mg, 500 µmol) was made to with phenanthrene-9-boronic acid (**5e**) (117 mg, 525 µmol) for 24 h using $Pd(PPh_3)_2Cl_2$ (10.5 mg, 15.0 µmol) as catalyst. In deviation from GP 1, the solvent of the filtrate was removed *in vacuo*, the residue was redissolved in DMSO (4 mL) and added dropwise to EtOAc (100 mL). The yellow precipitate was collected, washed with H_2O (5 mL), EtOAc (20 mL) and Et_2O (20 mL) and dried in vacuo. The product **6e** was obtained as yellow, amorphous solid (48.9 mg, 100 µmol, 20%); mp > 215 °C (dec.). – ^{1}H-NMR (600 MHz, DMSO-d_6): = 7.62–7.64 (m, 1 H, 7'-H), 7.68–7.70 (m, 1 H, 2'-H), 7.73–7.77 (m, 2 H, 3'-H, 6'-H), 7.87 (d, ^3J = 8.4 Hz, 1 H, 8'-H), 7.94 (s, 1 H, 10'-H), 8.05–8.09 (m, 2 H, 1'-H, 10-H), 8.12–8.14 (m, 2 H, 2-H, 6-H),

8.25–8.29 (m, 2 H, 11-H, 5-H), 8.37 (s, 1 H, 4-H), 8.69 (d, ^3J = 8.4 Hz, 1 H, 12-H), 8.87 (d, ^3J = 8.0 Hz, 1 H, 4'-H), 8.95 (d, ^3J = 8.0 Hz, 1 H, 5'-H), 9.25 (d, ^3J = 8.0 Hz, 1 H, 1-H), 9.41 (d, ^3J = 6.0 Hz, 1 H, 9-H), 10.13 (s, 1 H, 13-H), 10.32 (s, 1 H, 7-H). – ^{13}C-NMR (150 MHz, DMSO-d_6): = 120.5 (13-C), 122.7 (10-C), 122.7 (4'-C), 123.4 (5'-C), 123.7 (6-C), 125.3 (1-C), 125.7 (6a-C), 125.9 (8'-C), 125.9 (13b-C), 126.9 (12-C), 127.0 (6'-C), 127.1 (7'-C), 127.2 (2'-C), 127.3 (3'-C), 128.1 (10'-C), 128.8 (1'-C), 129.5 (4a'-C), 129.7 (8a'-C), 130.1 (4b'-C), 130.2 (4-C), 130.6 (2-C), 130.8 (10a'-C), 132.6 (5-C), 132.9 (11-C), 133.7 (4a-C), 134.6 (9-C), 135.1 (13a-C), 136.6 (9'-C), 137.3 (7-C), 138.8 (12a-C), 143.3 (3-C). – MS (ESI$^+$): *m*/*z* = 406 (100) [M–Br]$^+$. – El. Anal. for $C_{31}H_{20}BrN$ x 2 H_2O (522.44), calcd (%): C 71.27, H 4.63, N 2.68, found (%): C 71.39, H 4.13, N 2.87.

1.3 Determination of fluorescence quantum yields

Solutions were prepared for each measurement from stock solutions in a suitable solvent (MeOH for **6a**–**d**, $CHCl_3$ for **6e**; c = 1.00 mM). For experiments in different solvents, aliquots of the stock solution were evaporated under a stream of nitrogen and redissolved in the respective solvent. In general, absorption spectra were determined with a detection speed of 120 nm min^{-1} in a range from 275 to 450 nm and subsequently smoothed in the Origin software with the function "adjacent-averaging" (factor of 10).

For the detection of emission spectra the excitation and emission slits were adjusted to 5 nm. The detection speed was 120 nm min^{-1} and the detector voltage was adjusted between 500 V and 600 V depending on the fluorescence emission intensity. The spectra were smoothed with the implemented moving-average function by a factor of 5. The relative fluorescence quantum yields of the aryl-substituted naphtho[1,2-*b*]quinolizinium derivatives were determined under identical conditions, i.e. the same cuvettes were used and the measurements were performed at a constant temperature with the same settings on the spectrometer (detection wavelength, excitation wavelength, detector voltage, slit bandwidths, collection rate). Coumarin 1 (Φ_F = 0.73 in EtOH)[2] (for **6a** and **6b**) and Coumarin 153 (Φ_F = 0.38 in EtOH)[2] (for **6c**–**e**) were used as standards. The emission spectra were collected from solutions with Abs = 0.10 at the respective excitation wavelength. After integration of the fluorescence band, the relative fluorescence quantum yields were calculated according to Eq. 1.[3,4]

$$\phi_F = \frac{J_x(1-T_s)}{J_S(1-T_x)} \cdot \frac{n_x^2}{n_S^2} \cdot \phi_{F,S} \qquad \text{(Eq. 1)}$$

The subscripts “x” and “s” refer to the substance under investigation and a reference compound, respectively; $J = \int I_F(\lambda)d\lambda$ is the emission integral over the area of interest; T is the optical transmittance of the sample solution at the excitation wavelength, λ_{ex}; n is the refractive index of the sample or standard solution; however the quotient of the refractive indexes was neglected because it does not differ significantly from 1.

2 Additional spectroscopic data

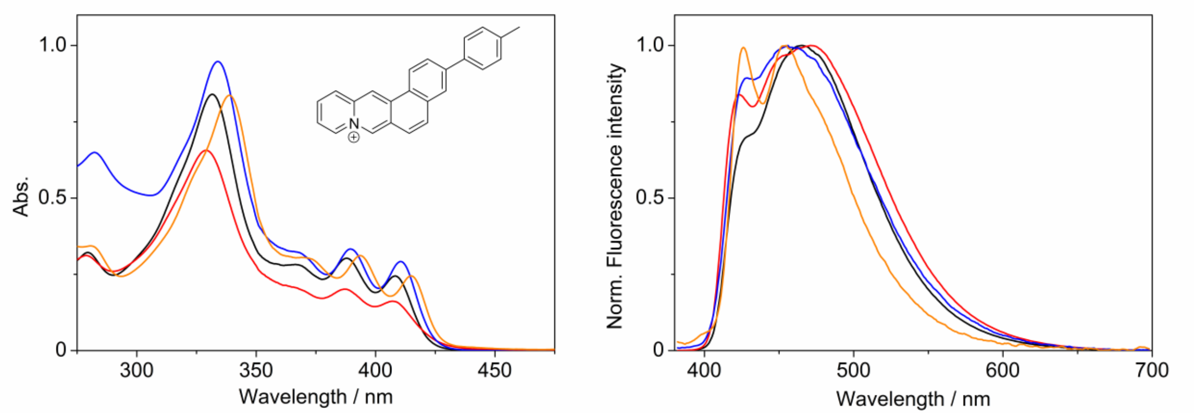

Figure S1: Absorption (left) and normalized emission spectra (right) of **6b** (c = 20 µM) in different solvents: H_2O (red), MeOH (black), DMSO (blue), $CHCl_3$ (orange).

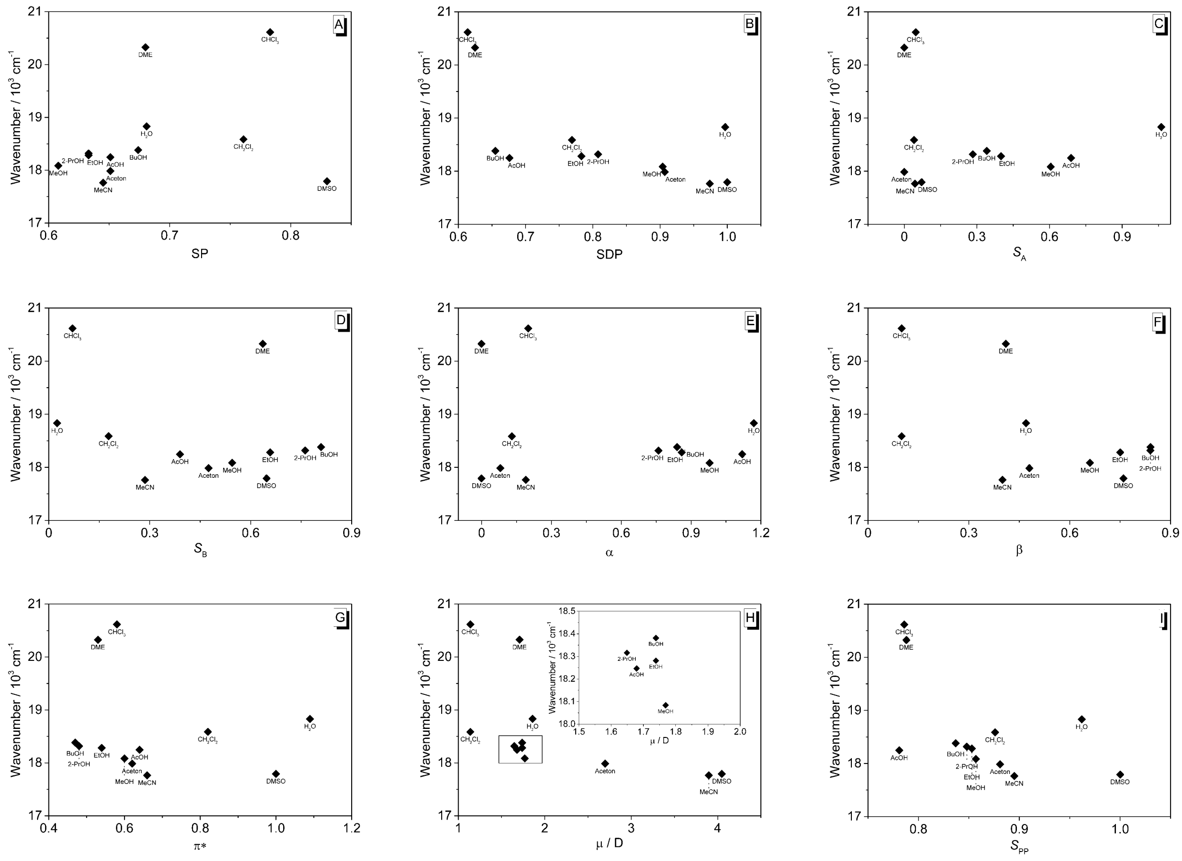

Figure S2: Plot of the emission maximum of **6e** in different solvents *versus* the polarizability, SP (A),[5] the dipolarity SDP (B),[5] the acidity, S_A (C),[6] the basicity, S_B (D),[6] the hydrogen bond (HB) donating properties, α (E),[6] the HB accepting properties, β (F),[6] the Taft parameter π^* (G),[6] the dipole moment, μ (H),[6] the dipolarity and polarizability S_{PP} (I).[6]

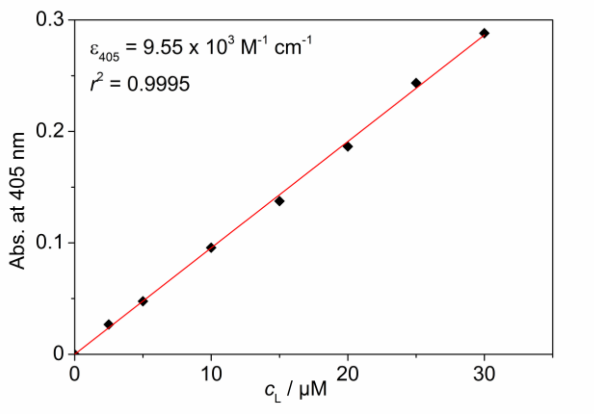

Figure S3: Plot of the absorbance of **6c** in H_2O at λ = 405 nm *versus* concentration.

3 ^{1}H-NMR spectra of derivatives 3, 4 and 6a–e

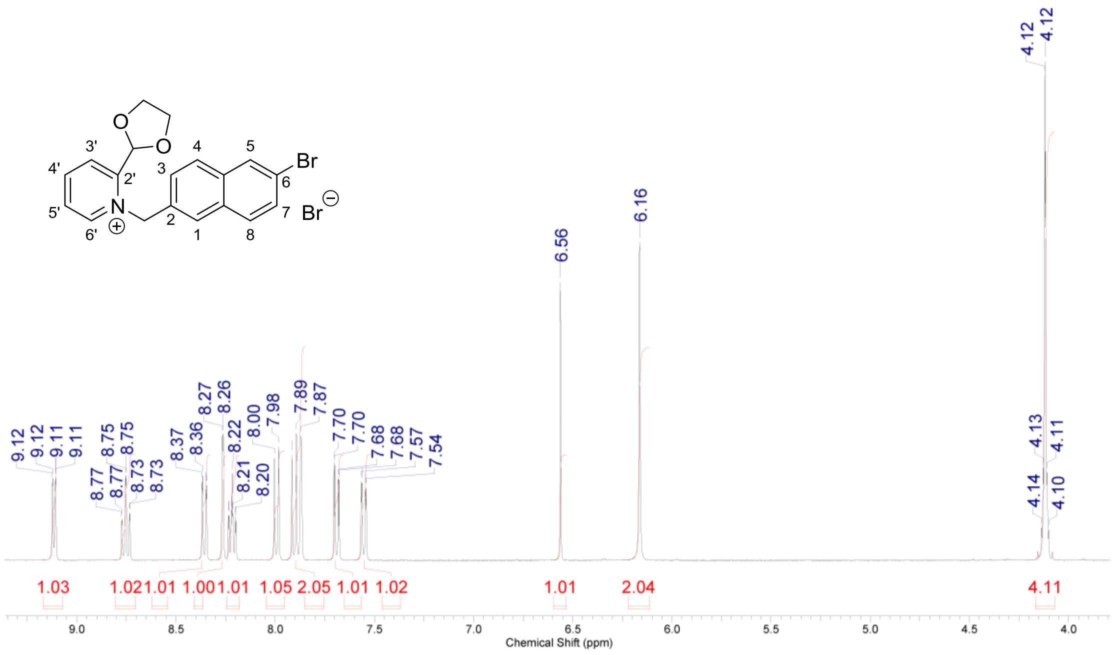

Figure S4: ^{1}H NMR spectrum of 1-[(6-bromonaphth-2-yl)methyl]-2-(1,3-dioxolan-2-yl)pyridinium bromide (**3**).

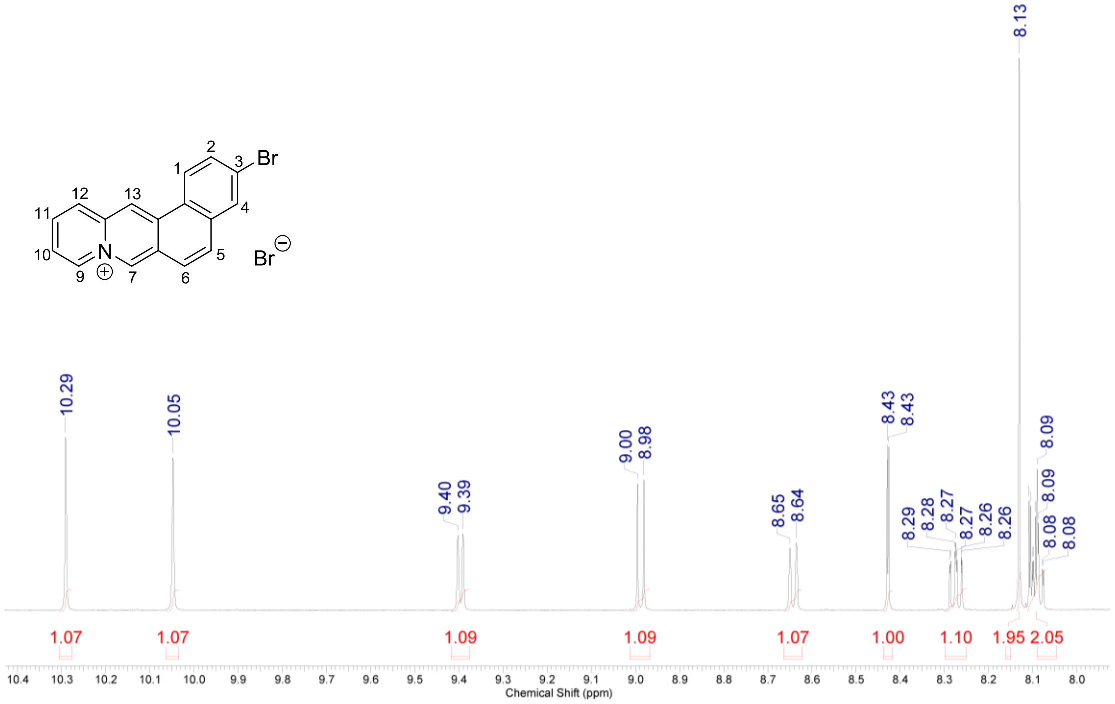

Figure S5: ^{1}H NMR spectrum of 3-bromonaphtho[1,2-*b*]quinolizinium bromide (**4**).

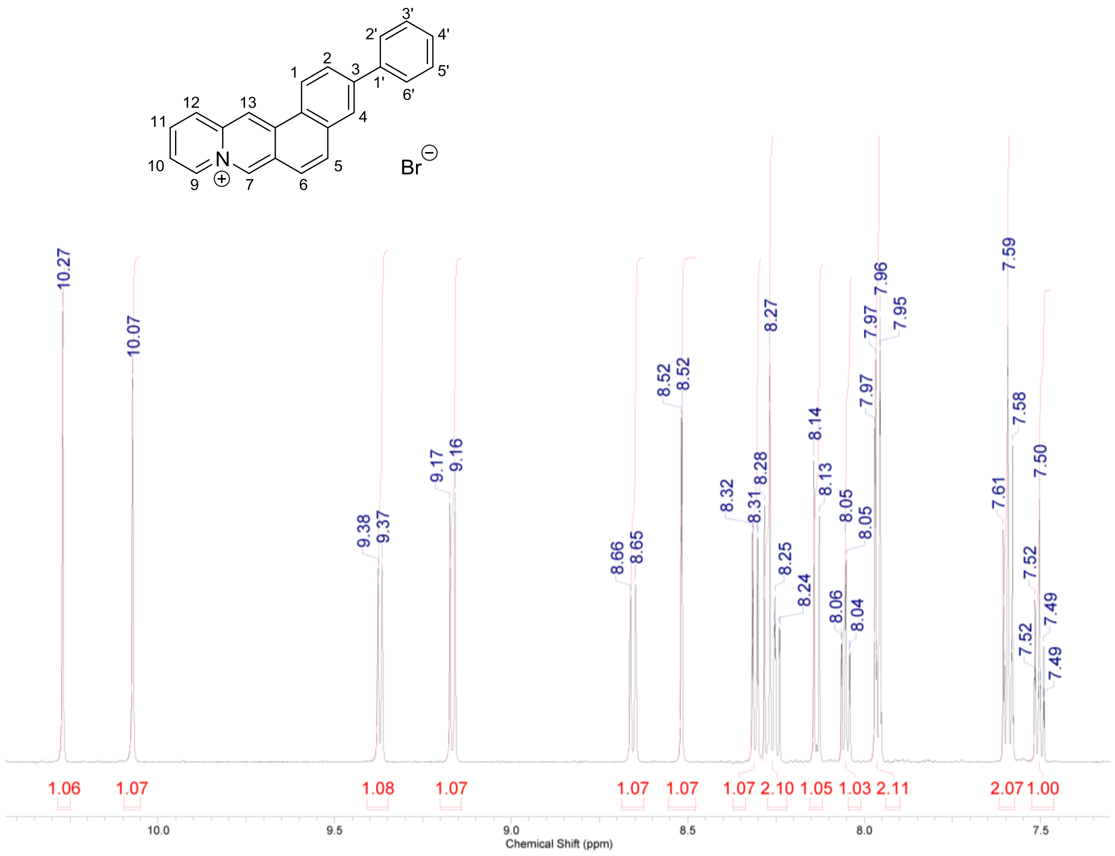

Figure S6: ^{1}H NMR spectrum of 3-phenylnaphtho[1,2-*b*]quinolizinium bromide (**6a**).

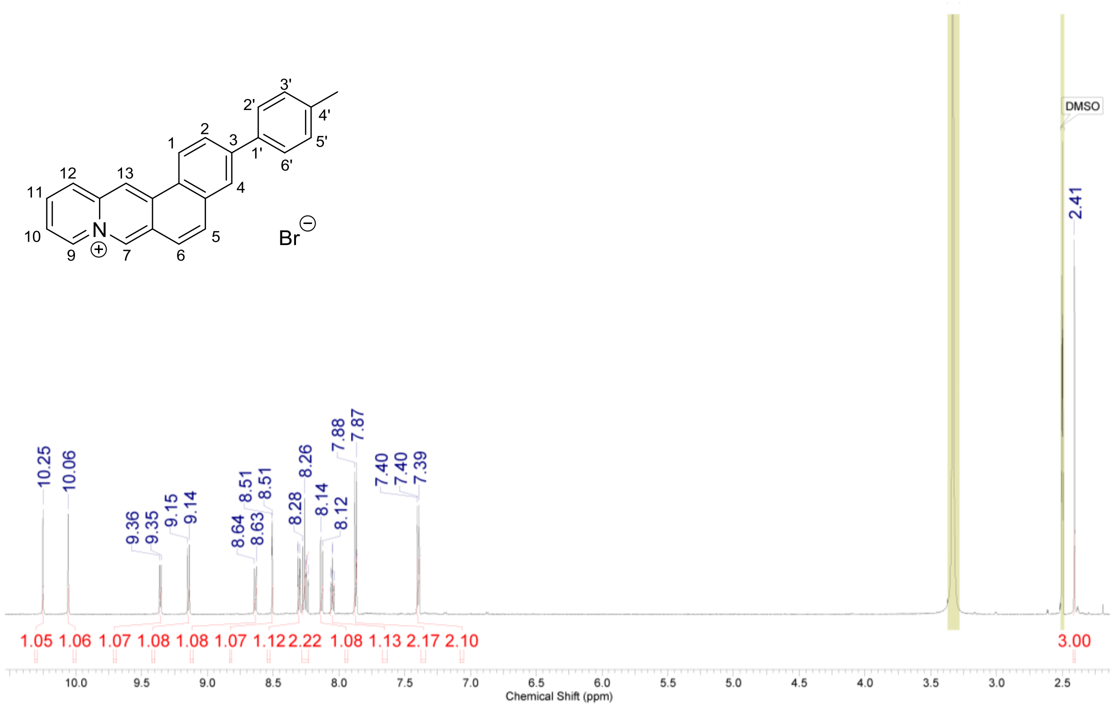

Figure S7: ^{1}H NMR spectrum of 3-(4-methylphenyl)naphtho[1,2-*b*]quinolizinium bromide (**6b**).

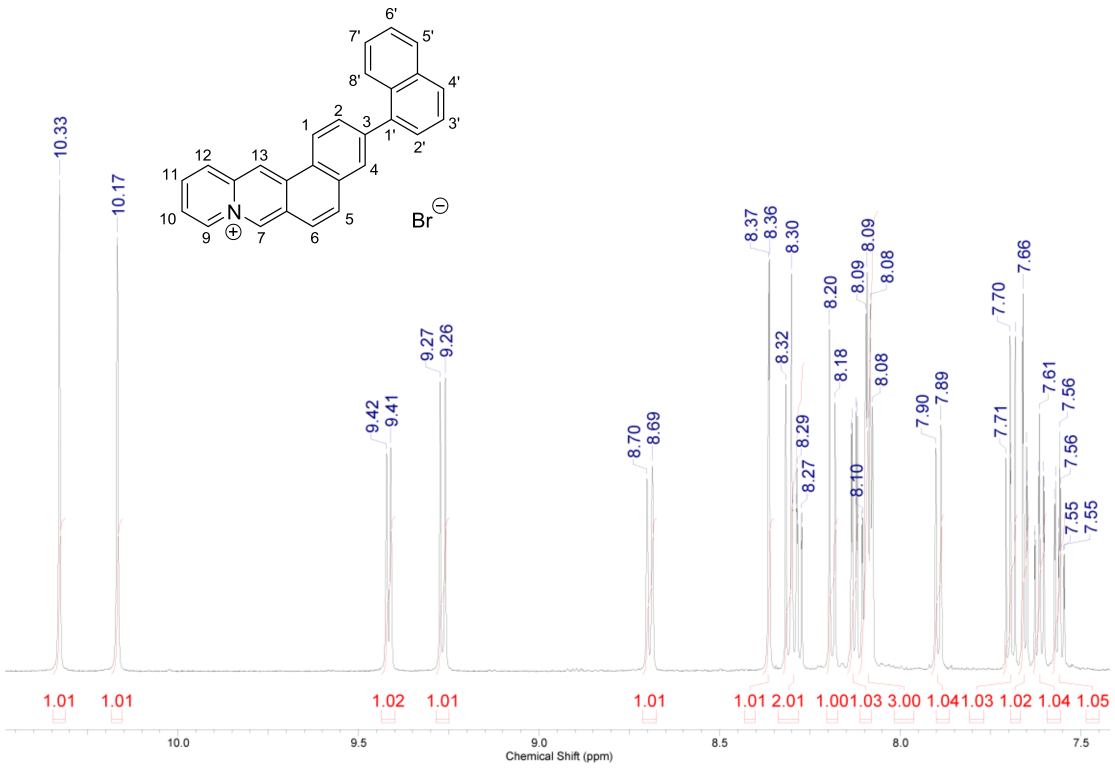

Figure S8: ^{1}H NMR spectrum of 3-(naphth-1-yl)naphtho[1,2-*b*]quinolizinium bromide (**6c**).

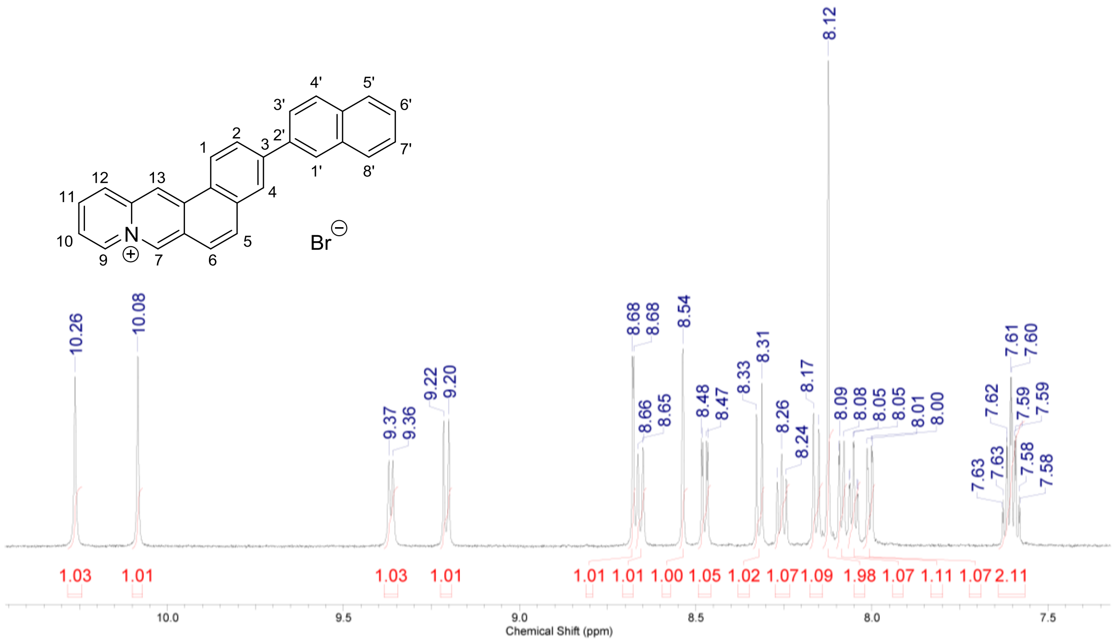

Figure S9: ^{1}H NMR spectrum of 3-(naphth-2-yl)naphtho[1,2-*b*]quinolizinium bromide (**6d**).

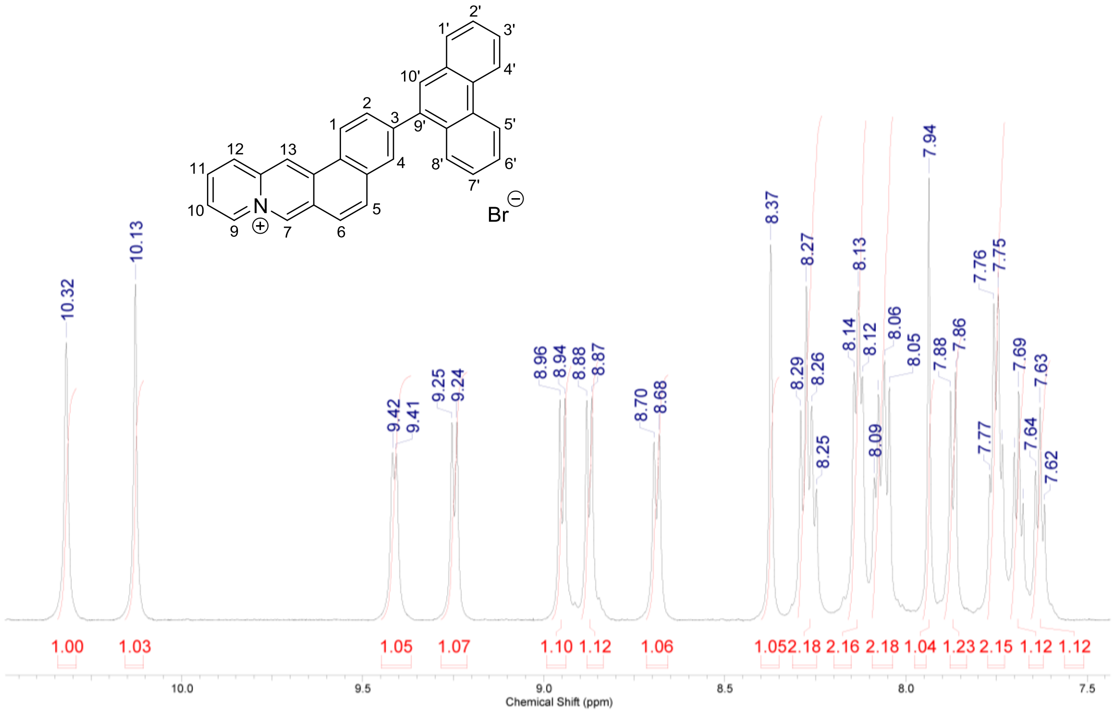

Figure S10: ^{1}H NMR spectrum of 3-(phenanthrene-9-yl)naphtho[1,2-*b*]quinolizinium bromide (**6e**).

4 References

[1] Tian, M.; Ihmels, H. *Synthesis* **2009**, *24*, 4226–4234.

[2] Jones, G.; Jackson, W. R.; Choi, C. Y.; Bergmark, W. R. *J. Phys. Chem.* **1985**, *89*, 294–300.

[3] Valeur, B.; Berberan-Santos, M. N. *Molecular fluorescence. Principles and applications*, 2nd ed.; WileyVCH: Weinheim **2012**.

[4] Crosby, G. A.; Demas, J. N. *J. Phys. Chem.* **1971**, *75*, 991–1024.

[5] Catalán, J. *J. Phys. Chem. B* **2009**, *113*, 5951–5960.

[6] Reichardt, C.; Welton, T. *Solvents and solvent effects in organic chemistry*, 4th, updated and enl. ed.; Wiley-VCH: Weinheim **2010**, and references cited therein.
